# Tolerance to baked milk in Brazilian children with persistent cow’s milk allergy

**DOI:** 10.1186/2045-7022-5-S3-P165

**Published:** 2015-03-30

**Authors:** Claudia P G Barbosa, Andrea A K F Gushken, Glauce H Yonamine, Ana Paula B M Castro, Antonio Carlos Pastorino, Cristina M A Jacob

**Affiliations:** 1 Unit of Allergy and Immunology, Child´s Institute, Department of Pediatrics, Faculdade de Medicina da Universidade de São Paulo, Sao Paulo, Brazil

## Objective

To identify among a group of patients with persistent cow´s milk allergy those who could tolerate the ingestion of extensively heated milk products (baked milk), and define if there are any different phenotypes between these two groups.

## Methods

Patients with cow´s milk allergy, over 4 years-old, were submitted to oral challenge with baked milk, from January to November, 2013, at Universidade de São Paulo, Brazil. The tested product was a muffin that contained 2,8 g milk protein and was baked at 350° F in an oven for 30 minutes. The challenge was made under physician supervision and at the first sign of allergy reaction it was discontinued, and the patient received the proper medical assistance.

## Results

Twenty eight patients underwent baked milk challenge. Six children were excluded from this trail (three could not eat the entire muffin and three tolerated baked milk and also unheated milk). From the 22 remaining patients, it was found a median age of 8y5m (4y10m to 13y11m), 5 were male. Eleven patients (50%) did not react to baked milk, while were unheated milk reactive.

The comparison of clinic features (age, sex and history of anaphylaxis), did not show any significant difference between the baked milk tolerant group and the baked milk reactive group. For Prick Test, there was significant difference to α-Lactoalbumin and to Casein, p 0,005 and 0,007 respectively. For laboratory findings, Milk IgE and Casein IgE presented significant difference between the two groups, p 0,049 and p 0,005 respectively.

## Conclusion

Through the identification of the patients that can tolerate baked milk, it is possible to improve quality of life for those who have cow´s milk allergy, being able to be set free in its diet products with baked milk. New studies with bigger samples are necessary to confirm the association of the casein as marking of risk patient to be reactor to the baked milk.

